# Care Coordination for High-Need, High-Cost Commercially Insured Patients: A Randomized Clinical Trial

**DOI:** 10.1001/jamanetworkopen.2025.11804

**Published:** 2025-06-24

**Authors:** O. Kenrik Duru, Jessica Harwood, Tannaz Moin, Sae Takada, Chi-Hong Tseng, Rintu Saju, Ella Lee, Anusha Fatehpuria, Carol M. Mangione

**Affiliations:** 1 Division of General Internal Medicine and Health Services Research, David Geffen School of Medicine at UCLA, Los Angeles, California; 2 Health Services Research and Development, Center for Healthcare Innovation, Implementation, and Policy, VA Greater Los Angeles Health Care System, Los Angeles, California; 3 Department of Medicine, Brigham and Women’s Hospital, Boston, Massachusetts; 4 VA Greater Los Angeles Health Care System, Los Angeles, California; 5 UCLA Jonathan and Karin Fielding School of Public Health, Los Angeles, California

## Abstract

**Importance:**

High-need, high-cost (HNHC) patients account for 5% of the US population yet represent nearly half of health care spending.

**Objective:**

To evaluate whether national care coordination could reduce health care cost and utilization in a commercially insured HNHC population.

**Design, Setting, and Participants:**

This national, 2-arm randomized clinical trial with intention-to-treat and instrumental variable analyses included patients aged 18 years or older who were defined as HNHC according to a proprietary model (in the top 5% of spend within a rolling 12-month claims utilization window and projected to remain in the top 5% over the subsequent 12 months). Patients were randomized from January 2018 to October 2019. Data were analyzed from January 1 to December 31, 2024.

**Intervention:**

Participants were randomized monthly 60:40 to telephonic care coordination from a registered nurse, including medication review, a barriers-to-care survey, addressing urgent coordination needs (eg, patient unable to fill prescriptions), development of a case management plan addressing identified clinical risk factors, and establishing an outreach time frame, or to the control group with usual care. The nurse contacted patients over the 60 days after enrollment until all risk factors included in the management plan were addressed.

**Main Outcomes and Measures:**

The main outcomes were mean monthly emergency department visits, inpatient hospitalizations, and total plan cost (medical and pharmacy) over 12 months following the index date, defined as the enrollment date for intervention participants or the randomly generated synthetic enrollment date for nonparticipants. Outcomes were examined separately among patients with diabetes.

**Results:**

The analytic sample included 93 379 HNHC patients with a mean (SD) age of 46 (12) years (54% female). In intention-to-treat analyses, there were no differences between groups in mean (SE) monthly emergency department visits (0.033 [0.001] for control vs 0.033 [0.001] for treatment; mean difference [SE], 0 [0]; 95% CI, −0.001 to 0.002; *P* = .69), inpatient hospitalizations (0.009 [0] for control vs 0.010 [0] for treatment; mean difference [SE], 0.001 [0]; 95% CI, 0-0.002; *P* = .06), or cost (total: $2507 [$32] for control vs $2568 [$26] for treatment; mean difference [SE], $60 [$41]; 95% CI, −$20 to $140; *P* = .14). In the instrumental variable analyses and in the subsample with diabetes, no evidence of statistically significant reductions in these outcomes were found.

**Conclusions and Relevance:**

In this randomized clinical trial of a national care coordination intervention, neither health care cost nor acute care utilization was reduced in the intervention group compared with the control group. The results emphasize the challenges of improving efficiency of care in a complex HNHC population with escalating health care costs.

**Trial Registration:**

ClinicalTrials.gov Identifier: NCT04415515

## Key Points

**Question:**

Can a national care coordination intervention reduce acute care utilization and health care cost in a commercially insured high-need, high-cost population?

**Findings:**

In this randomized clinical trial including 93 379 adults, there were no differences over 12 months in emergency department visits, hospital admissions, or total cost between patients randomized to the intervention or to usual care.

**Meaning:**

This large-scale care coordination intervention was ineffective in reducing cost or acute care utilization, which underscores the significant challenges of improving care efficiency in a complex high-need, high-cost population.

## Teaser

This randomized clinical trial evaluates whether a care coordination intervention reduces health care cost and acute care utilization among high-need, high-cost commercially insured patients.

## Introduction

A relatively small group of individuals often termed as high-need, high-cost (HNHC) represent a disproportionate share of US health care spending. Approximately 5% of patients account for nearly half of US spending on health care,^1^ many of whom have persistent high spending over several years.^2,3^ While there is no universal definition of HNHC, these patients typically have complex clinical, behavioral, and social needs, including multiple chronic diseases with or without functional limitations and require frequent emergency department visits and hospitalizations.^4,5,6,7,8^ This disproportionate spending on a small high-need subgroup is evident among Medicare-eligible adults as well as commercially insured patients younger than 65 years.^9,10^ Providing high-quality and cost-efficient care to HNHC patients represents a critical, unmet charge for clinicians, health systems, and payers and is a key health policy challenge.

Care coordination involves organizing patient care activities and sharing information across the entire patient care team to improve safety and efficiency. Numerous care coordination and management interventions for HNHC patients have been rigorously evaluated, with limited evidence of effectiveness.^4,11,12,13,14,15,16,17,18^ A meta-analysis of high-risk care management programs found no significant difference in patient utilization, cost, or mortality, although some studies demonstrated improvements in self-reported health status and patient satisfaction.^19,20,21,22,23^ Similarly, a review of care coordination among Medicare beneficiaries with chronic illnesses found that 13 of 15 coordination programs did not demonstrate hospitalization reductions.^24,25^ The Camden Coalition randomized clinical trial (RCT) evaluated an intensive 90-day multidisciplinary care coordination program involving home visits for HNHC patients and found no difference in 180-day readmissions.^26^ While there are reports of care coordination interventions that have reduced acute care utilization, most have been limited by small sample sizes (eg, <1000 patients), high attrition, and lack of randomization or unbalanced randomization.^4,27,28,29,30^

Our RCT of national care coordination for commercially insured HNHC patients implemented by a large insurer is, to our knowledge, one of the largest to date. The intervention was designed to provide whole-person care, simultaneously addressing medical, psychological, and social needs. Key outcomes included total health care costs (medical and pharmacy) and acute care utilization (inpatient hospitalizations and emergency department visits). Given that more US health care spending goes toward diabetes than any of 154 other studied health conditions, we also conducted analyses in a subsample of adults with diabetes.^31^

## Methods

### Study Design and Data Source

This pragmatic RCT included individual patients as the unit of analysis (trial protocol in Supplement 1). Patients were randomized by the insurer after they were identified as HNHC, defined as being in the top 5% of spend in a rolling 12-month claims utilization window and likely to remain in the top 5% of spend over the subsequent 12 months based on proprietary models developed by the insurer. We analyzed administrative claims data from January 1, 2014, to December 31, 2022, that included eligibility and medical and pharmacy claims as well as laboratory values from January 1, 2016, to December 31, 2022. Patients were randomized monthly 60:40 to treatment or control from January 2018 to October 2019. This RCT was approved as exempt by the UCLA institutional review board with a waiver of informed consent because it was not considered human-participant research, as data sent by the insurer and analyzed by UCLA were deidentified such that obtaining informed consent was not feasible. The study followed the Consolidated Standards of Reporting Trials (CONSORT) reporting guideline.

### Participants

Inclusion criteria were HNHC status, age 18 years or older, and enrollment in a fully insured commercial health plan at the time of randomization. Prerandomization exclusion criteria included pregnancy, prescription of infertility medications, dementia, and enrollment in a subset of plans not representative of the larger population, including plans for which the insurer provided administrative services only or plans assigned to a clinically activated accountable care organization.

This RCT was pragmatic in that patients in either study arm could enroll in the intervention at any time if they (1) were discharged from an inpatient facility and identified as having elevated risk for readmission based on a second proprietary algorithm or (2) were referred by a physician or self-referred. Thus, after randomization, we excluded patients in either arm who enrolled (agreed to participate) in the intervention prior to randomization (since their participation was independent of randomization status). We further excluded patients aged 65 years or older, patients with Medicare as their primary insurance, those qualified for competing clinical support interventions (eg, patients with cancer or advanced chronic kidney disease or kidney failure or organ transplant recipients), and those who left the health plan before the start of our study period (Figure).

**Figure.**
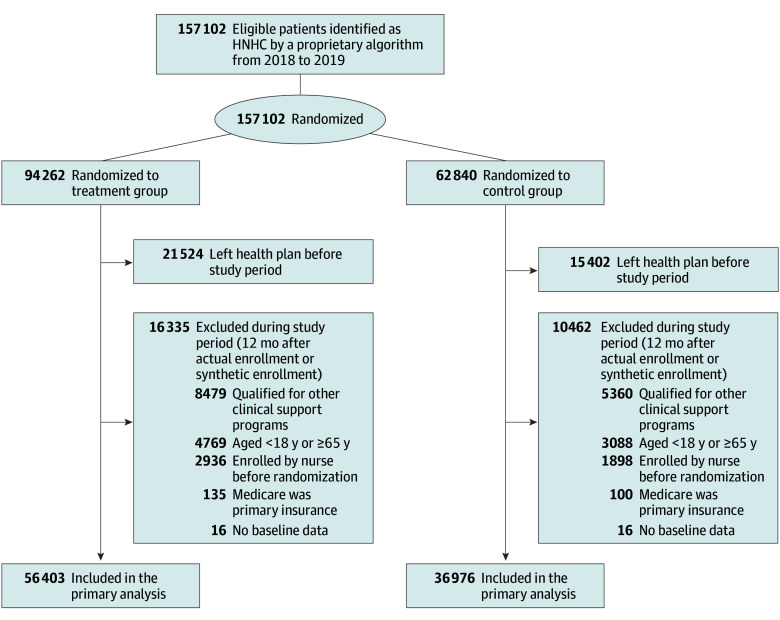
CONSORT Diagram HNHC indicates high-need, high-cost.

### Intervention

After randomization and subsequent confirmation of eligibility, patients assigned to the treatment group received an introductory preengagement message by mail or email followed by up to 4 attempted telephone calls from a registered nurse or, in rare cases, a nonclinical staff member if the nurse caseload was excessive. The telephone calls confirmed patient eligibility and gauged interest in participation. Participants who opted to enroll were asked to complete the following 5 intervention activities: (1) a self-reported medication review supplemented by claims data, (2) a clinical assessment to identify clinical and social risk factors, (3) addressing of any urgent care coordination needs (eg, unable to fill prescriptions following hospital discharge), (4) development of a patient-centered case management plan addressing identified clinical and social risk factors, and (5) establishment of the next outreach time frame, generally within 7 days. Patients were contacted regularly over the next 60 days until all clinical risk factors and/or needs from the patient-centered care management plan were addressed. Addressing these risk factors could include improving medication adherence through pharmacist consultations, completing medication reconciliation, improving clinical condition–based measures and outcomes, referral to smoking cessation, addressing psychosocial needs through attending office hours with behavioral health clinicians and/or social workers, and transitioning support following an inpatient hospitalization. If the nurse determined that case management would take longer than 60 days, the patient was referred for a higher level of case management that typically included engagement with the patient’s primary care practitioner and a more comprehensive assessment of risks for adverse health events, gaps in care, and barriers to care, including an increased focus on social determinants of health. Patients referred to this higher level of case management were included in the current analysis.

### Study Period and Intervention Enrollment Dates

We used the enrollment date to determine the index date for outcome ascertainment in the intervention group. Since this was not possible for nonparticipants, we randomly generated a synthetic enrollment date or index date for nonenrollees using the method described by Harvey et al.^32^ We defined the study window as the 12-month period after enrollment or synthetic enrollment given the time lag in implementing intervention-related activities.

### Measures

We measured study outcomes within the 12-month study period. We examined the following 3 coprimary outcomes: (1) mean monthly number of inpatient hospitalizations, (2) mean monthly number of emergency department visits, and (3) mean monthly total plan cost (medical and pharmacy). For the intention-to-treat (ITT) analyses, patients were compared by randomization status so that the regressor of interest was an indicator for randomization assignment to the treatment group (1) or the control group (0). For the instrumental variable analyses, we used the definition of engagement provided by the health insurer. Patients who satisfied any of the following criteria were considered as engaged: (1) an open patient dashboard that was being tracked by the registered nurse, (2) a completed barriers-to-care assessment, (3) enrollment in a clinical or disease management program for a specific condition (eg, diabetes), or (4) device reading (eg, continuous glucose monitor).

We included the following covariates as measured in prerandomization data: age group, sex, US geographic division, health plan type, and 19 comorbidity indicators.^33^ We also included a categorical variable for the year each person was randomized (2018 or 2019). Finally, for each of the coprimary outcomes, we included a baseline measure of that outcome over the 12 months before randomization. All cost measures were adjusted to 2022 US dollars using the consumer price index for all medical care services.

### Statistical Analysis

Data were analyzed from January 1 to December 31, 2024. For the primary ITT analyses of the full sample, we conducted multivariate linear regression analyses and adjusted for multiple comparisons (3 coprimary outcomes) by using an α level of .05/3 = .017. Regressions were repeated for the secondary analysis among the subsample of patients with diabetes.^34^ We also conducted secondary instrumental variable analyses on the same outcomes using randomization status as the instrument for engagement (an indicator for intervention engagement vs no intervention engagement). Interpretation of the ITT analyses was limited by low intervention uptake, as most patients randomized to the treatment arm did not participate. Furthermore, 5% of patients randomized to the control arm participated in the intervention, representing treatment contamination. The instrumental variable analyses addressed these challenges by modeling the potential effect of the intervention on the subgroup of patients who would participate in the intervention if they were randomized to the treatment arm but would not participate if they were randomized to the control arm, often called compliers.^35,36,37^ The complier average causal effect is an estimate of the intervention effect in this subgroup. We used a generalized method of moment estimation for the instrumental variable analyses with SEs that were robust to heteroskedasticity.

To address potential analytic biases related to secular trends, variability of the intervention over time, and incomplete capture of study outcomes, we conducted 9 sensitivity analyses (eTables 1-9 in Supplement 2). These included separate analyses for patients who were randomized in 2018 vs 2019, using the date of randomization rather than the enrollment date (or synthetic enrollment date) as the start of the study window and dichotomous measures for the study outcomes, limiting analyses to patients with continuous enrollment throughout the 12-month study period and to patients whose study window did not overlap with the COVID-19 pandemic (ie, March to December 2020), examining study windows of 18 or 24 months, and limiting analyses to a 6-month study window. We also examined study outcomes for an earlier iteration of the intervention delivered from August 2015 to December 2017. During this earlier period, the health insurer was not able to contact and enroll most eligible patients due to resource constraints, so this sample was less representative of the underlying population. Two-sided *P* < .05 was considered statistically significant. Database management was performed using SAS, version 9.4 (SAS Institute Inc). Statistical analyses were performed using Stata, version 17 (StataCorp LLC).

## Results

Between 2018 and 2019, the health insurer randomized 157 102 HNHC patients but subsequently excluded 63 723 who were identified as ineligible after randomization. Therefore, our analytic sample included 93 379 HNHC patients (mean [SD] age, 46 [12] years; 54% female and 46% male) (Figure). Patients randomized to treatment and control arms were similar in terms of baseline characteristics, including age, sex, comorbidities, number of months enrolled with the insurer during the study period, utilization, and cost (Table 1). Table 1 also shows the intervention engagement rate in each arm of the trial (treatment group: 56 403 patients [26%]; control group: 36 976 patients [5%]). Conversely, patients who engaged with the intervention were notably different from those who did not engage (eTable 10 in Supplement 2). Patients who engaged tended to be older with a greater likelihood of baseline comorbidities, including diabetes, hypertension, asthma, chronic obstructive pulmonary disease, hyperlipidemia, and osteoarthritis, compared with patients who did not engage. Patients who engaged also generally had slightly higher baseline costs.

**Table 1.** Sample Demographics by Randomization Arm

Characteristic	Participants^a^		
	Total (N = 93 379)	Control (n = 36 976)	Treatment (n = 56 403)
Age, mean (SD), y	46 (12)	46 (12)	46 (12)
Sex			
Female	54	54	54
Male	46	46	46
Months enrolled in study period, mean (IQR)	NA	9 (6-12)	9 (6-12)
Engaged and participated in the intervention	18	5	26
Baseline outcomes			
Months enrolled in baseline year, mean (IQR)	NA	9 (6-12)	9 (6-12)
Monthly inpatient hospitalizations, mean (SD)	NA	0.02 (0.07)	0.02 (0.07)
Monthly emergency department visits, mean (SD)	NA	0.06 (0.15)	0.06 (0.15)
Monthly plan cost, mean (SD), $			
Total	NA	3916 (6043)	3880 (5635)
Medical	NA	2909 (5916)	2863 (5494)
Pharmacy	NA	1007 (2093)	1017 (2338)
Comorbidities			
Diabetes	21	21	21
Arthritis	24	23	24
Asthma	14	14	14
Atrial fibrillation or cardiac dysrhythmias	18	18	18
Autism	<1	<1	<1
Cancer	33	33	34
Chronic obstructive pulmonary disease	9	9	9
Congestive heart failure	3	3	3
Dementia	1	1	1
Depression	20	20	20
Hypertension	41	40	41
HIV infection	3	3	3
Hyperlipidemia	38	39	38
Liver disease	9	9	9
Myocardial infarction or coronary artery disease	9	9	9
Osteoporosis	2	2	2
Schizophrenia	1	1	1
Stroke	5	5	5
Substance use	18	18	18

Abbreviation: NA, not applicable.

^a^ Data are presented as percentage of participants unless otherwise indicated.

In the primary ITT analyses, randomization to the treatment arm was not associated with changes in mean monthly emergency department visits (0.033 [0.001] for control vs 0.033 [0.001] for treatment; mean difference [SE], 0 [0]; 95% CI, −0.001 to 0.002; *P* = .69), acute hospitalizations (0.009 [0] for control vs 0.010 [0] for treatment; mean difference [SE], 0.001 [0]; 95% CI, 0 to 0.002; *P* = .06), or cost (total: $2507 [$32] for control vs $2568 [$26] for treatment; mean difference [SE], $60 [$41]; 95% CI, −$20 to $140; *P* = .14) compared with randomization to the control arm for the overall study sample over the 12 months following enrollment or synthetic enrollment (Table 2). Similarly, we did not find significant differences between arms in the subsample with diabetes (Table 3). In the secondary instrumental variable analyses, we did not find evidence of differences for those who engaged in the intervention (compared with those who did not engage) over the same 12-month period, representing the complier average causal effect for patients who likely would have taken up the intervention if they were randomized to the treatment arm but would not have taken up the intervention if they were randomized to the control arm (Table 4). The *F* statistic for our instrumental variable was 9944, suggesting that randomization was not a weak instrument for engagement. In the sensitivity analyses examining patients randomized in 2018, limited to patients with continuous enrollment during the 12-month study window, and examining outcomes over an 18-month window, we observed a statistically significant increase in inpatient hospitalizations for the treatment group, but this finding was not clinically significant (eTables 4 and 8 in Supplement 2). All other sensitivity analyses showed findings similar to those of the primary analyses.

**Table 2.** Multivariate Results Examining Cost and Utilization by Study Arm in the Primary Intention-to-Treat Analysis^a^

Outcome	Regression-adjusted mean (SE)		Difference, mean (SE) [95% CI]	*P* value
	Control	Treatment		
Inpatient hospitalizations^b^	0.009 (0)	0.010 (0)	0.001 (0) [0 to 0.002]	.06
Emergency department visits^b^	0.033 (0.001)	0.033 (0.001)	0 (0) [−0.001 to 0.002]	.69
Plan cost, $				
Total^b^	2507 (32)	2568 (26)	60 (41) [−20 to 140]	.14
Medical	1281 (28)	1310 (23)	29 (36) [−42 to 100]	.42
Pharmacy	1232 (13)	1253 (11)	20 (17) [−13 to 54]	.23

^a^ The analytic sample included eligible participants randomized between 2018 and 2019 (N = 93 379). Results are from linear regression. The regression covariate of interest was an indicator for randomization group (treatment vs control). Other covariates included sex, age group, geographic division, plan type, a categorical variable for the year the person was randomized (2018 vs 2019), 19 comorbidity indicators, and a baseline measure of the outcome (for utilization variables, the average monthly value of the outcome in the 12 months before randomization; for cost variables adjusted to 2022 US dollars, decile of average monthly outcome value for the 12 months before randomization).

^b^ Primary outcomes. Multiple comparisons adjustment: α of .05/3 = .017.

**Table 3.** Multivariate Results Examining Cost and Utilization by Study Arm for the Subsample With Diabetes in the Secondary Intention-to-Treat Subset Analysis^a^

Outcome	Regression-adjusted mean (SE)		Difference, mean (SE) [95% CI]	*P* value
	Control	Treatment		
Inpatient hospitalizations	0.011 (0.001)	0.011 (0.001)	0 (0) [−0.002 to 0.002]	.82
Emergency department visits	0.032 (0.001)	0.031 (0.001)	−0.001 (0) [−0.004 to 0.002]	.50
Plan cost, $				
Total	2354 (63)	2392 (51)	37 (81) [−121 to 196]	.64
Medical	1250 (59)	1259 (48)	9 (76) [−141 to 159]	.90
Pharmacy	1106 (17)	1132 (14)	26 (22) [−17 to 69]	.23

^a^ The analytic sample included eligible participants with diabetes randomized between 2018 and 2019 (n = 19 852). Results are from linear regression. Regression covariate of interest was an indicator for randomization group (treatment vs control). Other covariates included sex, age group, geographic division, plan type, a categorical variable for the year the person was randomized (2018 vs 2019), 18 comorbidity indicators, a baseline measure of the outcome (for utilization variables, the average monthly value of the outcome in the 12 months before randomization; for cost variables adjusted to 2022 US dollars, decile of average monthly outcome value for the 12 months before randomization), and the Diabetes Complications Severity Index composite score.^34^

**Table 4.** Multivariate Results Examining Cost and Utilization by Intervention Engagement Status in the Secondary Instrumental Variable Analysis^a^

Outcome	Regression-adjusted mean (SE)		Difference, mean (robust SE) [95% CI]	*P* value
	Not engaged	Engaged		
Inpatient hospitalizations	0.009 (0)	0.013 (0.002)	0.004 (0) [0 to 0.007]	.05
Emergency department visits	0.033 (0.001)	0.035 (0.003)	0.002 (0) [−0.006 to 0.009]	.68
Plan cost, $				
Total	2494 (33)	2775 (154)	281 (180) [−72 to 633]	.12
Medical	1275 (29)	1410 (136)	135 (157) [−174 to 443]	.39
Pharmacy	1228 (16)	1323 (66)	95 (79) [−59 to 250]	.23

^a^ The analytic sample included eligible participants randomized between 2018 and 2019 (N = 93 379). Results are from instrumental variable regression analysis (generalized method of moment estimation and SEs that are robust to heteroskedasticity) using random-assignment status (indicator for treatment vs control group) as an instrument for engagement (the covariate of interest, an indicator for intervention engagement vs no intervention engagement). Other covariates included sex, age group, geographic division, plan type, a categorical variable for the year the person was randomized (2018 vs 2019), 19 comorbidity indicators, and a baseline measure of the outcome (for utilization variables, the average monthly value of the outcome in the 12 months before randomization; for cost variables adjusted to 2022 US dollars, decile of average monthly outcome value for the 12 months before randomization).

## Discussion

This national, pragmatic randomized clinical trial of care coordination for 93 379 commercially insured HNHC patients that was implemented by a large national insurer did not observe effects on the coprimary outcomes of inpatient hospitalizations, emergency department visits, or total costs of care. Our findings were consistent in the ITT analyses overall and for the subsample with diabetes and in the instrumental variable analyses evaluating the complier average causal effect. As such, this intervention was not effective in reducing cost or acute care utilization either at the population level or in the subgroup that would be most likely to engage in the intervention if they were given the opportunity to participate. Of note, this was a relatively low-intensity intervention centered around a telephonic nurse intervention to identify unmet needs and facilitate clinical referrals. The absence of observed effects in the instrumental variable analyses suggests that strengthening this intervention will be relatively more important than increasing uptake in terms of targeted approaches to improve effectiveness.

These negative findings are consistent with much of the published literature in this field, but the large sample size of this trial, randomized design, and robust instrumental variable analyses address important literature gaps. There are a multitude of complex, logistical challenges associated with the creation and implementation of care coordination interventions for HNHC patients that may have contributed to the absence of a detectable intervention effect, including fragmentation of health care delivery, difficulty with accurately identifying patients who should be targeted for such an intervention, and a relatively narrow spectrum of intervention outcomes that are not necessarily patient centered.

The fragmentation of health care delivery in the US is an important consideration. For most commercially insured adults, care is typically fragmented among insurers, health systems, local practice and clinic sites, pharmacies, and community-based ancillary health services organizations. From the patient perspective, there are multiple practitioners involved in their care who may provide differing guidance at cross-purposes with one another.^38^ Over time, patients often switch health systems, usual sources of care, pharmacies, and practitioners, further amplifying instability and increasing the challenge for a single health care entity to create an effective program for HNHC patients. Prior interventions that attempted to institute system-level change were generally unsuccessful at decreasing cost of care among HNHC patients, potentially because they were too distal to patients and/or too limited in intensity.^4^ Identifying new, effective approaches that overcome system fragmentation and deliver extended, multilevel strategies to improve care efficiency for HNHC patients remains a significant challenge.

Precise, timely identification of patients who will be HNHC in the near future is another challenge, as recent intensive and expensive health care use or costs for these individuals regress to the mean. Interventions enrolling patients who are not truly HNHC over time will be less likely to show demonstrable effects on utilization and cost. While the current analyses relied on a proprietary prediction model, the literature examining nonproprietary prediction models shows that only 28% to 51% of HNHC patients remained HNHC after 12 months while others died or returned to non-HNHC status.^39,40^ Berkman and colleagues^4^ proposed a 3-part framework to identify HNHC patients that is tied to evidence predicting high follow-up utilization. This framework includes (1) the clinical or functional group (eg, multiple conditions, specific chronic conditions, and individuals younger than 65 years with disabilities), (2) high-impact behavioral and/or social risk factor needs (serious mental illness, substance use disorder, low socioeconomic status, and housing insecurity), and (3) prior high use and/or cost. A hybrid approach that combines quantitative prediction modeling and individual qualitative assessment, including socioeconomic and psychosocial circumstances, will be time-consuming and difficult but may be the best way to build such a predictive model for future interventions.^2^

Finally, utilization and cost outcomes may not capture the full benefits of care coordination interventions for HNHC patients. More work should be done to identify patient-centered measures of success as leaders in complex care delivery and patient advocates have recommended.^41,42^ Care management interventions should be routinely evaluated to assess expanded, holistic metrics beyond utilization and cost, including patient experience and satisfaction and health equity.^38^ A broadened approach may identify additional patient-centered measures of change, such as improved relationships and trust with care practitioners and staff, more care continuity, better self-reported health, or changes in social determinants of health that potentially represent milestones on a pathway to decreased cost and utilization over a longer time frame.

### Limitations

This RCT has limitations. The study had relatively low intervention uptake. However, our secondary instrumental variable analyses were designed to attenuate the effect of selection bias related to differential engagement in the intervention. We did not have details on the postrandomization exclusion of patients by the insurer, which may limit the generalizability of our findings. However, the intervention and control patients remain balanced on all baseline variables, indicating that these insurer exclusions did not lead to a biased study sample. We did not have access to clinical data to examine changes in management for study patients, and it was infeasible to assess process outcomes and program fidelity in this large, national intervention.

## Conclusions

In this RCT of a national care coordination intervention, neither cost nor acute care utilization was reduced. These null findings were observed in both primary ITT analyses and secondary instrumental variable analyses that examined a subset of patients more likely to take up the intervention when offered. Our results emphasize the significant challenges of improving efficiency of care in a complex HNHC population, which represents a significant driver of escalating health care costs in the US. Future care coordination programs for HNHC patients should maximize coordination across levels of the health care delivery system, use transparent and validated models to identify HNHC patients, and include a broader spectrum of patient-reported outcomes.

## Notes

Supplement 1.
Trial Protocol

Supplement 2. **eTable 1.** Multivariate Results Examining Cost and Utilization by Study Arm and Year **eTable 2.** Multivariate Results Examining Cost and Utilization by Study Arm, With Randomization Date Rather Than Engagement (or Synthetic Engagement) Date as the Index Date (n = 115 791) **eTable 3.** Multivariate Results Examining Cost and Utilization by Study Arm, With Dichotomous Outcomes (n = 93 379) **eTable 4.** Multivariate Results Examining Cost and Utilization by Study Arm, Among Patients With Continuous Enrollment Through the 12-Month Study Window (n = 52 049) **eTable 5.** Multivariate Results Examining Cost and Utilization by Study Arm, With a 6-Month Study Window (n = 92 838) **eTable 6.** Multivariate Results Examining Cost and Utilization by Study Arm, for Patients Randomized 2015 to 2017 (N = 187 791) **eTable 7.** Sensitivity—People Whose Study Period Did Not Overlap With the COVID-19 Pandemic (eg, Excluding March to December 2020; N = 58 466): Commercial RCT Regression Analysis Results in the 12 Months Postenrollment/Proxy, by Random Assignment Status **eTable 8.** Sensitivity—Commercial RCT Regression Analysis Results in the 18 Months Postenrollment/Proxy, by Random Assignment Status (N = 52 487) **eTable 9.** Sensitivity—Commercial RCT Regression Analysis Results in the 24 Months Postenrollment/Proxy, by Random Assignment Status (N = 53 208) **eTable 10.** Sample Demographics by Intervention Engagement Status

Supplement 3.
Data Sharing Statement
