# Supplementary material for: Care Coordination for High-Need, High-Cost Commercially Insured Patients: A Randomized Clinical Trial
Source: JAMA Netw Open. 2025 Jun 24;8(6):e2511804. doi: 10.1001/jamanetworkopen.2025.11804 (PMC12188368; doi:10.1001/jamanetworkopen.2025.11804)
Supplement: Supplement 1. — Trial Protocol [file jamanetwopen-e2511804-s001.pdf]

I. Purpose of the Study and Background

IIa. Purpose of the Study

Case management (CM) and care coordination (CC) programs were designed to partner with high-cost, complex, at-risk consumers to facilitate health care access and decisions that can have a dramatic impact on the quality and affordability of the consumer's health care. CM programs leverage a collaborative process of assessment, planning, facilitation and advocacy for options and services to meet an individual's health needs through communication and available resources to promote quality cost-effective outcomes.[1] CC is defined as a process of improving communication among the various medical professionals with whom patients come in contact and between these professionals and their patients (and their families).[1] In general, CM and CC programs show modest gains in quality of care.[2,3] However, programs with strong transitional care interventions that target high risk patients were found to lower hospital admissions.[2-5]

The current quality improvement study (QIS) was designed to determine if case management and care coordination (CM/CC) provided to commercially insured members identified via a proprietary administrative algorithms as being either 1) high-need, high-cost (i.e., identification in the top 5% of spend in the previous year and in the next 12 months) or 2) high inpatient risk for admission would significantly impact rates of acute inpatient admissions, 30 day all-cause re-admissions, outpatient emergency room (ER) visits, primary care physician (PCP) visits, and specialist physician visits. The entire intervention group will be eligible for the CM/CC program and will be compared to a control group which will only receive the CM/CC program if they are 1) identified as high risk for readmission upon discharge from the hospital, 2) are self-referred, or 3) are directly referred to the program by their physician.

The goal of the QIS is to 1) identify if the expansion of the CM/CC program to members identified as being at high risk would improve selected health outcomes, and 2) if the addition of a community health worker to the CM/CC model would improve selected health outcomes. This is important because the identified population is at high risk for future hospitalization. The two specific aims of the current QIS include:

a. *Challenge the current state via a pragmatic randomized controlled trial with health plan members as the unit of randomization*. Randomly allocate approximately 705,000 high-risk members into two groups over a four-year period. Compare rates of utilization including acute inpatient admissions, readmissions, ER visits, PCP visits, and specialist visits. This knowledge will allow decision makers to minimize uncertainty as they evaluate opportunities to expand the program.
b. *Investigate the comparative effectiveness of the approaches to improve health outcomes.* The identification of an improved approach to the expansion of the CM/CC program could lead to reduced complications and improved health outcomes.

The long-term aims of the proposed study are to disseminate the study's findings. The authors anticipate that the communication of positive study results would result in the expansion of the CM/CC model to the entire high-need, high-cost population and ultimately, lead to improved health outcomes for members with high costs and utilization.

II.b. Background

Each day individuals make decisions that impact their health and health care costs. The Centers for Disease Control and Prevention has identified that 50 percent of an individual's health status is determined by behavior — not genetics, environment or access. In response to these and similar findings, the CM/CC solution was designed by UHG as a clinically integrated and consumer-focused program of assessment, planning, facilitation and advocacy that leverages all available resources, including the health care team, the payer, and the community.

As a first step, it is important to define the rubric within which the programs of interest fall. The assignment of a rubric allows you to understand whether the intervention is tied to an event, a diagnosis, or a more general risk

state, as well as the nature and length of the intervention. The CM/CC program includes embedded case management and care coordination components designed to provide short-term and long-term, individualized consumer-centric approaches to managing the unique, costly and complex medical needs of high-risk members.

The Case Management Society of America defines case management (CM) programs as a collaborative process of assessment, planning, facilitation and advocacy for options and services to meet an individual's health needs through communication and available resources to promote quality cost-effective outcomes.[1] This definition encompasses needs for both acute and long-term care.[1] CM programs are generally long-term interventions focused on individuals at high-risk for a specific outcome. The results for geriatric CM programs suggest that they improve quality measures important to vulnerable elders and may reduce hospitalizations among those at highest risk for hospitalization.[4] Within the CM/CC program, a consumer is either enrolled in the Stage 1 program, or the Stage 2 program if the member has a high level of clinical, psychosocial or behavioral needs.

The Stage 1 program focuses on value pillars (right care, right medications, right provider, and right lifestyle) to minimize disease progression and future spend. As part of Stage 1, a Registered Nurse (RN) educates and empowers the consumer to address care opportunities and achieve desired outcomes. Stage 1 cases may require multiple contacts; however, they do not meet criteria for promotion to Stage 2.

The Stage 2 program is available to highly complex members and/or members that require specialty focus by Certified Case Managers. Stage 2 focuses on value pillars to facilitate an appropriate plan of care with physician and consumer to address utilization and clinical issues. A proprietary scoring tool identifies the need for Stage 2 at the time of initial referral to Stage 1 to ensure continuity of management by a single Registered Nurse (RN).

There is no well-established definition for care coordination (CC); however, it is generally defined as a model that involves a process of improving communication among the various medical professionals with whom patients come in contact and between these professionals and the patients themselves (and their families).[1] CC programs are generally short-term interventions focused on a specific event. In general, CC programs show modest gains in quality of care.[2,3] However, programs with strong transitional care interventions that target high risk patients were found to lower hospital admissions.[2,3,5] While there are numerous studies regarding care coordination among Medicare and Medicaid populations,[2,3] there is a paucity of research focused on the commercially insured population,[5] particularly in the new Individual Public Exchange populations. The CM/CC model also includes embedded care coordination strategies designed to proactively monitor and manage hospitalized patients immediately after their discharge to ensure a safe transition to home.[5] Patients identified for this activity are considered as being at high risk for readmission due to their underlying illness, length of hospitalization, or other complex discharge plan.[5] Consumers are contacted either during or soon after hospital discharge and calls focus on helping the consumer understand his or her discharge instructions, completing medication reconciliation, ensuring the consumer has a follow-up appointment scheduled with a Primary Care Physician (or other appropriate physician), and other interventions that reduce the risk of readmission.

The program involves the process of identifying high-risk or at-risk consumers with acute and chronic healthcare needs that include unexpected catastrophic occurrences, as well as the proactive management of anticipated clinical needs. Members are eligible for the program if they meet one of the following criteria:

- Discharged from an inpatient facility and identified as being at high risk for readmission.
- Identified via physician referral or self-referral as someone who may benefit from care coordination (e.g., experiencing a health issue that requires extra attention)
- Determined to be "high risk" by via other methods.

Case managers encourage wise health care decisions by providing members with expert advice and clinical resources. The member's case manager is available to answer questions about health concerns, educate members on available resources, and inform them of other services that may be helpful. Members experience continuity in their care by connecting with the same case manager from the start of a health event until it is completed.

The scope of the program extends throughout the continuum of care and includes the treating practitioner, consumer (or caregiver) education and engagement, and consumer self-management. The program is designed to support professionally licensed staff to objectively assess, plan, implement, monitor and evaluate the efficiency and appropriateness of care for participating consumers enrolled in the program. RN assessment areas include but are not limited to: current health status and history, treatment plan, resources needed, psychosocial status, health literacy, safety concerns, and functional limitations. Program services are delivered telephonically and through specific consumer mailings. Members with behavioral health needs are referred to appropriate entities per contractual arrangements.

Case management nurses engage both consumers and physicians as needed throughout the case management process. The nurse case manager works directly with the member to support their physician's treatment plan, provide education and coordinate services. The program is designed to complement the physician's treatment plan, reinforce their instructions as provided and offer support to the member to make healthful lifestyle choices. It is not intended to diagnose or treat, and it is not a substitute for the treating physician's professional, medical advice. Periodically, CM/CC Medical Directors may contact the member's physician(s) if it is identified that their patient has been non-adherent with their prescribed treatment regimen or lacks interventions suggested by evidence-based medicine.

In 2014, the CM/CC program was available to commercially insured individuals across lines of business. Approximately 30,000 members participated in the program in 2014. The current study was designed to determine the following:

> ***Objective 1:*** if the CM/CC program would statistically significantly impact health outcomes for commercial members identified as being high-need, high-cost (the QIS is powered to answer this primary objective), and
>
> ***Objective 2:*** if the addition of a Community Health Worker to the care team would substantially impact health outcomes for Individual Public Exchange members identified as being high-need, high-cost (the QIS is not powered to answer this secondary objective).

It is important to note that throughout the study period, the CM/CC program will continue to be available to all commercial health plan members similar to previous years, including the control group in the current QIS, who are 1) discharged from an inpatient facility and identified as being at high risk for readmission and 2) identified via physician referral or self-referral as someone who may benefit from care coordination.

III. Criteria for Subject Selection

III.a. Baseline Studies

III.b. Number of Subjects: As of July 2015, there were 4.5 million program eligible members. Among the eligible population 102,014 (point prevalence of 2.28%) were identified via a proprietary administrative algorithm as being high-need, high-cost (i.e., identification in the top five percent of spend and utilization in year 1 and year 2, based on member characteristics in Year 1). Since the inclusion algorithm will be refreshed monthly for 12 months, modelling based on 2014 data suggests that approximately 200,000 unique members will be identified as being at high-need, high-cost, and included in the randomization frame, over the first 12 months of the QIS.

III.c. Gender of the Subjects: Participants are expected to be 60.1% female.

III.d. Age of the Subjects: The age range of the sampling frame will be limited to ≥18 years of age. Participants are expected to have an average age of 47.5.

III*. e. Racial and Ethnic Origin:* Racial and Ethnic data are only self-reported for 3.5% of the commercial population: 20.7% African American, 1.9% American Indian/Alaskan Native, 4.8% Asian, 64.5% White, Non-Hispanic, 6.6% Hispanic, 0.0% Native Hawaiian or Other Pacific Islander, 1.2% Other, 0.3% Declined to Answer, and 0.0% Unknown to Member or Uncoded The distribution of race/ethnicity of the high-need, high-cost population is expected to mirror the commercial population.

III. f. Inclusion Criteria: The commercial Fully Insured population members in all states are represented in the initial population prior to condition identification. This is also inclusive of the Individual Public Exchange business, part of Health Care Reform, also known as ObamaCare. The age range of the sampling frame will be 18 or greater years of age. Members must also be actively enrolled in the health plan as of the date of randomization. Study participants will be identified via proprietary administrative algorithm as being at high-need, high-cost.

III. g. Exclusion Criteria: The population sampled will exclude pregnant women, individuals prescribed medications for infertility, members with evidence of dementing disorders, and members under the age of 18 years old. Members in the following products and plans will also be excluded:

- legacy ASO groups (populations for which the insurer provides administrative services only),
- assignment to a clinically activated Accountable Care Organization (ACO)

Additional exclusions include members indicated as “do not contact “ for program outreach, , and members not actively enrolled in the health plan as of the date of randomization.

III. h. Vulnerable Subjects: The eligible population does not include prison inmates or employees of the health insurer. The population sampled will exclude pregnant women or individuals prescribed medications for infertility. Members with evidence of dementing disorders and members under the age of 18 years old will be excluded from the QIS as a proxy for members with limited cognitive function or decisional incapacity.

IV. Methods and Procedures

IV.a. Methods and Procedures

IV a. 1. Overall Strategy: The proposed QIS leverages a pragmatic randomized controlled trial design with the qualified member as the unit of randomization. Pragmatic studies of health services allow healthcare to occur in natural settings.[7,8]

The primary hypothesis for the **referral** for the **RN Only** model is that the utilization rates among the control population identified via the standard referral process (i.e., 5-10% of population qualified for Stage 1 and Stage 2 intervention due to high-risk discharge, self-referral, or MD-referral) is not different than the utilization rates among the intervention population identified by the new referral process (i.e., 100% of population qualified for the Stage 1 and Stage 2 intervention due to high-need, high-cost status). The current QIS is powered to answer this primary objective.

Ho_1: $[Y_{\text{standard referral process with CM/CC}} - Y_{\text{new referral process with CM/CC}}] = 0$

The secondary hypothesis for the **referral** for the expanded **RN+CHW** model is that the utilization rates among the control population identified via the standard referral process with the enhanced CM/CC model (i.e., 5-10% of population qualified for Stage 1 or Stage 2 intervention due to high-risk discharge or self-referral or MD-

referral) is not different than the utilization rates among the intervention population identified by the new referral process (i.e., 100% of population qualified for the Stage 1 or Stage 2 intervention due to high-need, high-cost status). The current QIS is not powered to answer this secondary objective.

Ho_2: $[Y_{\text{standard referral process with CM/CC}} - Y_{\text{new PSU referral process with enhanced CM/CC}}] = 0$

The secondary hypothesis for **inpatient referral** for the **RN Only** is that the utilization rates among the control population identified via the standard referral process with the enhanced CM/CC model (i.e., 5-10% of population qualified for Stage 1 or Stage 2 intervention due to high-risk discharge or self-referral or MD-referral) is not different than the utilization rates among the intervention population identified by the new referral process (i.e., 100% of population qualified for the Stage 1 or Stage 2 intervention due to high risk for PSU status).

Ho_3: $[Y_{\text{standard referral process with CM/CC}} - Y_{\text{new inpatient referral process with CM/CC}}] = 0$

***IV. a. 2*. Justification of Study Design**

IV. a. 2. Justification for a pragmatic design: Pragmatic, or naturalistic designs, are designed to evaluate how a technology works in normal or usual practice and take into account patient and provider variations and can range from experimental to quasi-experimental studies. Pragmatic experimental designs retain random allocation to treatments to minimize bias, but offer fewer restrictions in how patients are recruited and followed after randomization, thus increasing external validity and generalizability.[7,8] Pragmatic experimental designs have been used previously to evaluate strategies to promote the adoption of low-risk public health recommendations, such as CMS's evaluation of the Health Support pilot (i.e., Chronic Care Improvement Program)[9] or the National Health Service's (NHS) evaluation of the impact of fecal occult blood test (FOBT) screening for colorectal cancer[10].

Generally, experimental designs are structured so that recruitment precedes randomization in order to evaluate the efficacy of an intervention; however, randomization occurs before recruitment (i.e., enrollment) in pragmatic methodologies which are designed to evaluate the effectiveness of an intervention.[12,13] The use of the pragmatic design balances the goals of internal and external validity.[14]

IV. a. 3. Sample Size: The study was powered for acute inpatient admissions, the most restrictive outcome, using the sample size estimation for trials of recurrent events in SAS. A baseline study of patients meeting the high-need, high-cost criteria suggests that a total sample size of 171,454 (85,727 per treatment arm) would provide the study with >= 80% power to detect the following intent to treat differences between treatment groups at an alpha=0.05 over the first 12 months of the four year study:

- a 2.5% reduction in acute inpatient admissions
- a 2.5% reduction in 30-day all-cause readmissions
- a 2.5% reduction in emergency room visits
- a 2.5% increase in primary care physician visits
- a 2.5% increase in specialist physician visits

Given a treatment effect of a 20% reduction in utilization for the as-treated population, an average of 10.6 months of at-risk exposure, an estimated program engagement rate of 20% of the qualified population with an average of 6.5 months of engagement in the program, a sample size of 85,727 individuals in the program treatment arm was used to power the most restrictive outcome (i.e., acute inpatient admissions) using preliminary data. In other words, the study's original planned sample size of approximately 200,000 members for the initial 12-month period, including both treatment arms, was sufficient to allow decision makers to assess if the CM/CC intervention could improve the targeted outcomes in the qualified population by year. The larger

cumulative sample sizes, included in years 2-4 of the study also address the salient business need for continuous quality improvement – the foundation of a QIS.

IV. a. 4. Rationale for the study endpoint: The study endpoint is defined as documentation of utilization including acute inpatient admissions, 30-day all-cause re-admissions, outpatient emergency room (ER) visits, primary care physician (PCP) visits, and specialist physician visits using administrative claims data. These outcome measures align with National Committee on Quality Assurance's (NCQA) Healthcare Effectiveness Data and Information Set (HEDIS) quality measures[15], Centers for Medicare & Medicaid Services' (CMS) Five Star Quality Rating System for Medicare Advantage Plans[16], and prior analysis of the effectiveness of care coordination and case management programs[1-5], represent significant costs and indicators of complications as well as represent important factors needing additional research as it relates to the care of patients.

IV. a. 5. Justification for using administrative data: Access to and analysis of medical. behavioral, and pharmacy claims and notification data, including diagnosis codes, revenue codes and procedure code level detail is required to summarize, compare and document appropriate inclusion and exclusion criteria are met as well as utilization rates including inpatient admissions, emergency room visits and physician visits for the purpose of determining the effectiveness of the CM/CC program. The use of this administrative data aligns with methods used in published reports (e.g., HEDIS and Five Star) [15,16] on outcomes and research conducted on care coordination and case management program outcomes.[1-5]

IV. a. 6. Study limitations and alternative design

The primary limitation of the proposed study design is related to misclassification of high-need, high-cost status (i.e., identification in the top five percent of spend and utilization in the previous year and in the next 12 months) based on the prospective, proprietary administrative algorithm. This limitation could be addressed via the expansion of the current study to include outreach for all members in the top five percent of spend and utilization in the previous year. In prior comparative validity studies (N= 149,011), the use of all members in the top five percent of spend and utilization in the previous year as a predictor of top five percent of spend and utilization in the next 12 months resulted in a sensitivity of 100% and a PPV of 36%. However, the application of this alternative design has a significant financial burden since only 36% of members in the top 5% of cost in the previous year prospectively remain in the top 5% in the next year. The identification and stratification of qualified members is a critical component of the success of the program. With any case management program, cost effective allocation of limited case management resources can only be achieved if organizations can identify a small number of high-risk members with low false positive rates.[6] While the sensitivity and specificity of the predictive models are important, positive predictive validity (PPV) must be the primary focus. The prospective score allows for the identification of a smaller number of individuals with a higher sensitivity and higher PPV; thereby, allowing for the cost effective allocation of limited CM/CC resources. The same argument for the cost-effective allocation of limited resources applies to the new high inpatient risk algorithms.

The second limitation of the proposed study design relates to the following three scenarios where the control population may receive CM/CC: 1) the member is identified as being at high risk for readmission upon discharge from the hospital, 2) the member is directly referred to the program by their physician, or 3) the member self-refers to the program. Based on an April 2015 baseline study, it was estimated that this issue will only impact 5-10% of the control group. These two scenarios will result in some "contamination" of the effect in the control group resulting in the lack of a “pure” control group to determine program impact. Participants in both treatment arms are receiving differing levels of the intervention. This limitation could be addressed by limiting care coordination and/or case management to the intervention population. However, this would deprive members who are facing an imminent health situation (either a high-risk discharge or physician determined risk) from receiving the Stage 1 program. Furthermore, both treatment arms also have access to other care or disease management programs, for conditions such as cancer, chronic kidney disease, end-stage kidney disease, or organ transplant.

A third consideration is related to the evolving nature of the care coordination and case management program that will be delivered to members during the course of the study. As a part of continuous quality improvement, the program team may make changes to materials and interventions, and additional interventions may be added. The research team will assess the magnitude of any operational or clinical changes and appropriately inform the review board using established procedures.

Lastly, the proposed QIS was designed as a pragmatic study to evaluate the comparative effectiveness of two low-risk public health approaches to deploy the CM/CC program as they occur in natural settings. The goal of the study is to identify the optimal "real world" approach so that it can be leveraged across the entire population. As such, the QIS could not practicably be carried out without a waiver of informed consent. While a more general experimental design (recruitment followed by randomization) would allow for the documentation of informed consent for all participants, this design would also have external validity concerns. Furthermore, individuals in both treatment arms will receive the CM/CC intervention if they meet the criteria highlighted in the second limitation.

IV. a. 7. Randomization

Eligible participants will be randomly allocated into two groups. The sponsoring organization will own the randomization process. A unique random number will be generated for each member. The members will be randomly allocated each month to each arm. Restriction (e.g., blocking, stratification, matching) will be leveraged as required.

IV. a. 8. Interventions by Treatment Arm

***Intervention Group*:**
As described in above, 100% of the members randomized to the intervention treatment arm each month will be eligible to receive the CM/CC program. All eligible members are loaded into software to determine if the member is operationally qualified for outreach (e.g., not identified on 'do not call' list, currently enrolled in health plan, not qualified for other programs, valid telephone number, etc.). Based on an initial capacity study, it was originally estimated that CM/CC staff would only have capacity to manage approximately 20,000 unique members annually. However, operational capacity was increased to 35,718 unique members in 2018.

Members who are qualified for the program will receive a pre-engagement message either by mail or email. The pre-engagement message introduces CM/CC services to the member. The message also informs the member that they will receive a call about the program or that they could place a call to the inbound RN Case Manager phone line to discuss services.

There are two methods for outbound telephonic outreach that are used within the program.

1. A non-clinical staff specialist initiates the first contact with the member leveraging auto-dialer technology. Upon contacting the individual, the staff specialist validates that the individual is the member of interest and transfers the member to a registered nurse (RN). If the member is unable to speak for an extended period, the staff specialist schedules an appointment with the member and the RN at the member's convenience. If the member's spoken or preferred language is not English, the staff specialist will engage an external translator service, "Language Line Solutions" and will document in the member's case record the primary language for subsequent outreach. If necessary, the RN will also leverage "Language Line Solutions".

An RN Case Manager will directly dial the member's phone number and attempt contact. Once contact is made, a similar process is followed as above. The RN Case Manager will also offer to immediately begin working with the member to identify and reduce clinical risks.

The method used is based on RN Case Manager capacity given daily needs of existing case load and incoming referral volume. If the RN Case Management team has adequate capacity they will perform the direct dial method, which is preferred. If capacity is limited, the CM/CC program will leverage the auto-dialer and non-clinical staff specialist as the first point of contact.

If the member does not agree to participate, or if during the initial outreach call it is determined by the staff specialist that the member is not clinically eligible for the program, the program referral is rejected and the appropriate reason is documented. Additionally, the member can be excluded from additional calls in the future via an Opt-Out process if requested by the member.

If the member is not reached during the initial outreach call, a second call will be placed within 5 business days. Two additional outreach attempts will be attempted before the “No Contact Letter” letter is mailed to the member. After 30 days, the process of attempted outreach repeats.

Stage 1 Program – Active 11/01/2015 to 10/31/2019
Upon successful telephonic contact with the member, the RN provides the member with an overview of the Stage 1 intervention, advises the member of their rights and responsibilities, provides the member with their direct phone number (with private voice mail) and hours of operation, provides the member with the 24 hour, general helpline number (available to all plan members), confirms the name and address of the member’s primary managing physician(s), and confirms verbal agreement by the member to participate in the program. During the first call, the RN also engages in the following activities: 1) completes a self-reported medication review (supplemented with administrative data if applicable), 2) completes the Barriers to Care Adult Clinical Assessment of current conditions for the member focused on risks for adverse health events, gaps in care and barriers to care, , 4) addresses any urgent care coordination needs (e.g., member is unable to fill prescriptions following discharge from hospital), 5) develops a consumer-centric case management plan, and 6) informs the member of the next outreach timeframe. The initial assessment process outlined above may require multiple phone calls over the course of several days depending on the unique scenario. If a member misses a scheduled appointment, the RN will send the Missed Appointment Letter. In cases where a member is newly discharged from the hospital, the Discharge Assessment will be completed in place of the Barriers to Care Assessment (which may still be used at a later time). The Discharge Assessment was developed to help the RN Case Manager quickly identify and reduce re-admission risks. The Discharge assessment focuses on medication changes/risks, ensuring follow-up with a provider within 7 days of discharge or per the discharge instruction timeline, symptom management, access to DME, and other common post-discharge needs.

The second contact generally occurs within seven days or less. In general, the maximum timeframe of outreach is 30 days after the last contact with the member. Additional outreach may occur as needed at the nurse’s discretion depending on closure of gaps in care. If during the initial call or subsequent calls, an interpreter is needed to ask questions, complete assessments or cover educational mailings, the clinician makes use of the “Language Line Solutions” interpreter service and notes on the member’s case record their primary spoken language. If necessary, the initial Barriers to Care Adult Clinical Assessment can be updated. Furthermore, subsequent inpatient discharges can retrigger the capture and reprioritization of inpatient-related post-discharge care coordination opportunities since this is an event driven process.

Members are contacted at minimum monthly until all clinical risks and opportunity (gaps in care) are closed. Clinical risks are prioritized via the consumer centric case management plan. Clinical risk reduction focuses on improving medication adherence, medication reconciliation, improving condition-based measures and outcomes, addressing psycho-social needs, and intensive transition of care support following an inpatient admission. RN Case Managers refer cases to MDs or Pharmacists for case consultations as needed. MDs maintain ‘open office hours’ with Behavioral Health clinicians for Behavioral Health case consultation, when needed. MD’s will also coordinate with local health plan Chief Medical Officers and treating providers to develop customized plans for addressing members with high clinical risks who are difficult to engage. Social workers are also available to RN Case Managers to identify resources to address barriers to care. In cases where members cannot be reached, the RN Case Manager may perform a review of available clinical data

within the system for the members. If clinical risks are identified, a referral will be made to the MD to evaluate for peer-to-peer engagement with the member's primary care provider to socialize identified risks. CM/CC RN Case Managers may also refer members to additional services such as help finding a Behavioral Health Provider, Behavioral Case Management services via Life Solutions and smoking cessation via Quit For Life. A clinical risk is considered addressed when (1) an identified gap in care has been closed through change in treatment or behavior achieved by communication with member/provider (2) an identified gap in member education is addressed by the RN and member demonstrates understanding (3) the intervention is assessed and determined there is no gap and (4) when all appropriate steps have been taken (i.e. exhausted) but the gap is unable to be resolved.

During program management, members may be referred to Transplant Resource Services, the End Stage Renal Disease program, the Chronic Kidney Disease program, the Cancer Support program or the Heart Failure Disease Management program. When a member is referred to these programs they will also be transferred to the program and no longer directly serviced by the CM/CC program. Results for these programs, for both intervention and control groups, are included in the aggregate RCT results.

Members are actively managed based on the determined outreach schedule until they (1) leave insurance, (2) expire, (3) refuse to continue to participate in the program, (4) are lost to follow-up or (5) graduate the program due to clinical risks being resolved Graduating members will be re-enrolled following a subsequent high-risk acute inpatient discharge or direct referral from the member or the member's physician.

Stage 2 Program – Active 11/01/2015 to 10/31/2019
At any time, if the RN determines that the ongoing management of the member will take longer than the approximate 60-day intervention period for Stage 1 based on open gaps in care (measured via a proprietary scoring tool) and the combined clinical knowledge of the RN and Medical Director, the member may be promoted to more intense case management via Stage 2. If the member is identified for promotion to Stage 2, the RN contacts the member and provides them with an overview of the Stage 2 process, advises the member of their rights and responsibilities, notifies the member that they will receive the "Stage 2 Welcome Letter" which includes the patient rights and responsibilities as well as the Advanced Directives Brochure, and documents agreement by the member to participate in the program. The Stage 2 protocols for members who decline mirror those used in Stage 1.

After enrollment in Stage 2, the identified primary managing physician(s) is mailed a "Physician Welcome Letter" indicating that their patient has been enrolled in the Stage 2 program. The letter provides a general overview of the Stage 2 program and notifies the provider that nurse case managers and/or medical directors may contact them to support the provider's treatment plan and coordinate services. Upon promotion to the Stage 2 program, the RN addresses any urgent care coordination needs and completes the Comprehensive Barriers to Care Adult clinical assessment or the new Barriers to Care Adult clinical assessment which is tied to the earlier assessment; however, the comprehensive version has more extensive required elements to provide a more holistic view of the member's risks for adverse health events, gaps in care and barriers to care.

CHW CM/CC – Active 11/01/2015 to 7/1/2017
In selected markets for defined time periods, members randomized to the intervention arm also received an enhanced version of the CM/CC intervention that included in-home care management support from non-clinical Community Health Workers (CHW). A CHW was anticipated to be helpful to ensure that this population understood the general health insurance process, understood the vital importance of establishing a relationship with a Primary Care Physician (PCP), and was assisted with community resources as necessary in order to optimize their health care experience.

In general, the outreach protocol for this enhanced CM/CC intervention mirrored the outreach protocol described previously. However, there were a few differences. The primary difference was that the CHW performed the outreach call and scheduled a home visit with the member if they resided within the CHW's service area. In addition, each member was assigned to a Community Care Team which was comprised of an

on-site CHW, a telephonic RN, and other telephonic specialists as needed. The Community Care Team was accountable for end-to-end integrated person-centered care management of all members to whom they were assigned. All members were assigned to one on-site CHW. Following contact with the CHW, individuals identified with more complex needs were also assigned to a telephonic clinician from the Community Care Team for longitudinal interventions.

As members were enrolled in the program, the Community Care Team also ensured that each member was assigned a PCP, which may have been a specialist, if they had not yet chosen one or wished to change providers. Each member was strongly encouraged and steered towards the optimal use of the PCP as the medical home for community-based health and preventive services. As this link was established, the team involved the PCP in the plan of care (POC) development process and reinforced their treatment plan in accordance with the evidence-based clinical guidelines that form the foundation of the program.

The CHW tracked the status of all scheduled home visit attempts. In the case of an unsuccessful home visit, the CHW made three call attempts to reschedule the home visit. A field visit was attempted on members who were unable to be reached by phone by the CHW. During the home visit, the CHW completed the Access to Care questionnaire and the PHQ-9 survey. The information documented by the CHW included but was not limited to: primary care physician visits, transportation needs, medication concerns, and home health and durable medical equipment needs. If the member was recently hospitalized, the CHW completed the Post Hospitalization assessment.

If during the initial outreach call by the CHW, it was determined that the member was not within the service area, or if member refused a home visit, but agreed to talk over the phone, the CHW completed the Access to Care questionnaire and the PHQ-9 survey during the initial outreach call. If the member was not able to talk immediately, a follow-up telephone call was scheduled to continue the conversation.

During subsequent telephonic contact with the Community Care Team clinicians, the assigned RN may have also completed the Adult Core assessment or the SF-12 survey based on clinician judgment. Members were managed longitudinally until three months after they were no longer identified as eligible for the program.

***Control Group*:** It is important to note that throughout the study period, the CM/CC program will continue to be available to all commercial health plan members (including the control group in the current QIS) who are 1) discharged from an inpatient facility and identified as being at high risk for readmission and 2) identified via physician referral or self-referral as someone who may benefit from care coordination. It is expected that approximately 5-10% of the 100,000 members randomized to the control group will receive the CM/CC intervention. Furthermore, engagement in other programs will be monitored in order to determine if stratification is necessary since both treatment arms also have access to other care or disease management programs, such as Transplant Resource Services, the End Stage Renal Disease program, the Chronic Kidney Disease program, the Cancer Support program, or the Heart Failure Disease Management program.

IV. b. Data Analysis and Data Monitoring

Primary Data Analysis: All analyses will be based on administrative data. In order to address claims lag, the administrative data will be pulled four months following the end of each intervention period.

Univariate analyses (standardized differences) will be used to test for baseline differences between the treatment groups. Any statistically significant differences in baseline characteristics will be controlled for in the multivariable models assessing the study outcomes. Unless otherwise noted, all tests will be two-sided tests with alpha= 0.05. Model diagnostics will be utilized to evaluate the need to address outliers.

The primary study outcomes include the following:

- a 2.5% reduction in acute inpatient admissions
- a 2.5% reduction in 30-day all-cause readmissions
- a 2.5% reduction in emergency room visits
- a 2.5% increase in primary care physician visits
- a 2.5% increase in specialist physician visits

Utilization metrics will be compared between intervention groups at 12, 24, 36 and 48 months following randomization using a repeated measures Poisson or negative binomial regression model. Zero-inflated models will be utilized if appropriate. The Cox proportional hazards test will be used for all cause 30-day readmission. Results will be reported in aggregate, as well as stratified by year of randomization.

Secondary Data Analyses: The study will also explore numerous secondary outcomes that were not powered, but that are important for continuous quality improvement – the primary goal of a QIS. Examples include the following:

- Total cost of care: Utilization and cost will be evaluated for all service categories to identify hypotheses for continuous quality improvement.
- Variable cohort definitions:
    - A "once qualified, always qualified" definition will be used to evaluate the long-term impacts of the program after a member is no longer identified by the monthly refresh of the algorithms. This mirrors a more academic approach to the measurement of outcomes.
    - An "operational" definition will include members until 120 days after they are no longer actively identified by the monthly refresh of the algorithms. This view is designed to drive continuous quality improvement.
- Health plan type: The impact of the program among the Individual Exchange population compared to the traditional Fully Insured population will be evaluated.
- Disease: The comparative effectiveness of the program by disease will be evaluated to drive continuous quality improvement and to identify hypotheses for new program offerings. .
- Access to Other Programs: Qualification for other programs will be monitored in order to determine if stratification is necessary by program qualification for other programs since both treatment arms also have access to other care or disease management programs, such as Transplant Resource Services, the End Stage Renal Disease program, the Chronic Kidney Disease program, the Cancer Support program, or the Heart Failure Disease Management program.

Study Timeline: The proposed timeline for the QIS includes the following dates & activities:

10/01/2015 IRB Submission
10/08/2015 Program effective in tech platform
10/19/2015 Dialer team stages referrals
11/01/2015 **Intervention begins**. Members start receiving outreach
12/31/2015 1st monthly operational metrics report available
01/31/2016 1st monthly operational leading indicator report available
01/31/2016 1st monthly outcomes report (notifications) available
05/31/2016 1st monthly outcomes report (claims) available
06/01/2016 On-site CHW model expanded to 12 states
12/31/2016 Individual exchange plans end except for NY and VA
07/01/2017 CHW intervention ends
10/01/2017 Randomization moved from 50/50 percentage split to 40/60
09/01/2018 Engagement of inpatient referrals begin
10/31/2019 **Intervention ends**

03/31/2020 Final Study Results available.
04/30/2020 QIS Ends

IV. c. Data Storage and Confidentiality

All study investigators, required IT, and clinical operations staff performing member program management will have access to demographic and medical claims data for heath plan members as part of their daily work activities.

Participant demographic and medical claims data are accessed as necessary for daily operations. In accordance with our Risk Management approach to security implementation, numerous policies, procedures, and technical controls have been selected to manage access to systems and information. Home based employees are required to connect via a secure Virtual Private Network (VPN) which ensures all data are encrypted and not susceptible to interception. Secure Transmission Standards have also been selected and corresponding controls have been implemented to ensure the confidentiality, integrity and availability of electronic PHI transmitted via public networks. Controls utilize appropriate encryption and authentication or equivalent means to protect the transmitted information.

In order to perform the randomization, exclusion, mail notification and analytic process, the list of identified individuals with limited identifier and demographic data will be provided by the health insurer to the implementing program. Participant demographic data will be provided to external address and mailing vendors for the purpose of locating and verifying contact information needed for member outreach (telephone or mailing) and for mailing member and provider notification letters. The release of this information is managed via the Data Release Governance process.

The Security Guidance on Encrypted Methods of Delivery protocol outlines the approved methods of delivery for sending protected and/or confidential information outside of the insurer. Approved methods include the following Secure E-mail, Transport Layer Security, secure FTP through the External Customer Gateway, Winzip 9.1 (password must be encrypted in a separate e-mail), Roxio 8.0 CD encrypted burning, encrypted secure USB drive, and encrypted Connect:Direct/NDM.

IV. d. Transition from Research Participation

Members actively participating in the CM/CC program at the end of the 12-month pilot will continue to be managed until the closure reasons outlined in <u>*IV. a. 8. Interventions by Treatment Arm*</u> are documented.

V. Risk Benefit Assessment

***V. a.* Risk Category**

The risk category for the proposed QIS is **Minimal Risk**. The probability and magnitude of harm or discomfort anticipated in the research is not greater than those ordinarily encountered in daily life.

In 2014, the CM/CC program was available to over 4.5 million commercially insured individuals. Approximately 30,000 members participated in the program in 2014. The current study was designed to determine the impact of the expansion of the CM/CC program to members identified as being at high risk, as well as the impact of the addition of a Community Health Worker to the care team.

Case management nurses engage both consumers and physicians as needed throughout the case management process. The nurse case manager works directly with the member to support their physician’s treatment plan, provide education and coordinate services. The program is designed to complement the physician’s treatment plan, reinforce their instructions as provided and offer support to the member to make

healthful lifestyle choices. It is not intended to diagnose or treat, and it is not a substitute for the treating physician's professional, medical advice.

V. b. Potential Risk

Due to the fact that the claims identification rules do not accurately identify high-need, high-cost status 100% of the time, there is a possibility that a member who will not have increased cost and utilization in the next year will receive initial outreach and evaluation.

V. c. Protection against Risk

The CM/CC program is not intended to diagnose or treat, and it is not a substitute for the professional, medical advice of the member's treating physicians. The program is designed to complement the member's physician's treatment plan, reinforce their instructions, and offer support for healthful lifestyle choices.

It is important to note that throughout the study period, the CM/CC program will continue to be available to all commercial health plan members (including the control group in the current QIS) who are 1) discharged from an inpatient facility and identified as being at high risk for readmission and 2) identified via physician referral or self-referral as someone who may benefit from care coordination. This allows members who are facing an imminent health situation (either a high-risk discharge or physician determined risk) to receive the CM/CC solution.

This possibility of an erroneous notification of eligible status is described and mitigated by the content of the QIS procedures and the member's ability to follow up with their physician. Members may disenroll from the CM/CC program at any time.

V. d. Potential benefits

The benefits of the current QIS include an increased opportunity to educate and empower more high-risk consumers to address care opportunities and achieve desired outcomes. The hypothesis is that the expansion of the CM/CC intervention to the qualified population will better support the member's physician's treatment plan, reinforce their instructions, and offer support for healthful lifestyle choices; thereby, leading to improved health outcomes.

V. e. Alternatives to Participation

Alternative avenues include physician follow-up, available Internet and printed materials, currently available care coordination, case management, and disease management programs as well as national organizations that support individuals with specific diseases. Both treatment arms also have access to other care management programs, such as 24 hour Nurseline, Transplant Resource Services, the End Stage Renal Disease program, the Chronic Kidney Disease program, the Cancer Support Program, or the Heart Failure Disease Management program.

VI. Subject Identification, Recruitment and Consent/Assent

VI. a. Method of Subject Identification and Recruitment

The proposed QIS leverages a pragmatic randomized controlled trial with the member as the unit of randomization. Pragmatic studies of health services allow health care to occur in natural settings.[7,8,12,13] Generally, experimental designs are structured so that recruitment precedes randomization in order to evaluate the efficacy of an intervention; however, randomization occurs before recruitment (i.e., enrollment) per the pragmatic methodology. As such, the pragmatic design evaluates the effectiveness of an intervention.[12,13]

Introduced by Schwartz and Lellouch in 1967, the use of pragmatic experimental designs balances the goals of internal and external validity.[8,14]

Recruitment: Per the pragmatic design, all eligible members will be randomized to one of the two treatment arms. As such, there is no recruitment per se. Per Figure 1, all eligible members will have some level of exposure to the CM/CC intervention. As a part of the program engagement effort, individuals will have the ability to passively consent to participate in the program via their decision to enroll in the disease management program when contacted by a CM/CC staff or their decision to read the written material. However, it is important to note that this process does not represent informed consent to participate in the QIS. As described in the human subjects section, a waiver of informed consent will be secured for the QIS.

VI. b. Human Subjects

Pragmatic designs, which allow health care to occur in natural settings, are an important tool in the effort to evaluate the effectiveness of new technology. However, pragmatic designs do raise ethical questions within the context of quality improvement studies. The arguments for formal documentation of IRB review or exemption and the need for a formal waiver of informed consent were recently highlighted in the medical literature in response to an Office for Human Research Protection investigation of an experimental quality improvement study that was described in the New Yorker and the New York Times, as well as in several medical journals.[17-20] While not technically required, these steps would allow UHC to document that we were very thoughtful in our use of a pragmatic design to answer an important topic related to health care operations. In addition, the process would allow us to share results with the larger medical community via publication; however, publication of study findings is not the primary purpose of the current study.

Since the current QIS includes Individual Public Exchange health plans, the proposed project should meet the following criteria for IRB exemption from the Common Rule (under 45 CFR 46.101(b)(5)):

> "Research and demonstration projects which are conducted by or subject to the approval of department or agency heads, and which are designed to study, evaluate, or otherwise examine:
>
> (i) Public benefit or service programs; (ii) procedures for obtaining benefits or services under those programs; (iii) possible changes in or alternatives to those programs or procedures; or (iv) possible changes in methods or levels of payment for benefits or services under those programs."

However, the implementer will pursue formal IRB review for the current QIS due to the study's prospective nature and the unique approach to randomization and consent inherent in the pragmatic design.

The current study should also meet the four requirements for a waiver of informed consent. These requirements are:

1. The intervention involves no more than minimal risk to the individuals.
    a. The CM/CC program implemented in the current QIS was designed to support national recommendations for care. The literature suggests that care coordination and case management interventions similar to those described in the current study, can improve clinical outcomes in patients.[1-5]

2. The waiver will not adversely affect the rights and welfare of the subjects.

    a. All eligible members identified as persistent super utilizers will receive some level of access to the CM/CC program based on more limited eligibility and qualification criteria. However, all eligible and qualified members in the treatment group of the QIS will have access to a CM/CC program.

b. Randomization is the fairest method to allocate members to the two treatment arms since the comparative effectiveness of the two approaches is unknown at this time and CM/CC resources are limited.

c. Individuals still have the ability to passively consent to participate via their decision to engage in the CM/CC nurse phone call or read the written materials. However, it is important to note that this process does not represent informed consent to participate in the QIS.

d. Pragmatic designs have been used previously to evaluate strategies to promote the adoption of low-risk public health recommendations. CMS applied the SRCD design to evaluate the Health Support pilot (i.e., Chronic Care Improvement Program).[9] The National Health Service (NHS) used the design to evaluate the impact of fecal occult blood test (FOBT) screening for colorectal cancer.[10]

3. The QIS could not practicably be carried out without a waiver of informed consent.
   a. The current QIS was designed to evaluate the comparative effectiveness of the two approaches as they occur in natural settings. The goal of the study is to rapidly identify the optimal "real world" approach so that it can be leveraged across the entire commercial population.
   b. A more general experimental design (recruitment followed by randomization) would have external validity concerns.
   c. The process of informed consent for the current QIS would by definition act as a tailored intervention; therefore, biasing the results of the study to the null.

4. Whenever appropriate, the subjects will be provided with additional pertinent information after participation.
   a. If approved, the final results of the current QIS will be communicated to all enrolled commercial members.
   b. The National Committee on Quality Assurance's publishes the Healthcare Effectiveness Data and Information Set (HEDIS)[15] quality measures each year highlighting the care of health plan members, including inpatient and outpatient utilization rates, as well as 30-day readmissions.

VI. c. Cost to the Subjects

There is no cost to participants or providers for the proposed QIS.

VI. d. Payment for Participation

There is no payment to participants or providers for participation in the proposed QIS.

References

1. Centers for Medicare and Medicaid Services. Coordinating Care for Medicare Beneficiaries: Early Experiences of 15 Demonstration Programs, Their Patients, and Providers. May, 2004
2. Peikes et al. Effects of Care Coordination on Hospitalization, Quality of Care, and Health Care Expenditures among Medicare Beneficiaries. JAMA. 301(6): 603-618. 2009
3. Coleman EA, Parry C, Chalmers S, et al, The care transitions intervention: results of a randomized controlled trial. Arch Intern Med. 2006; 166(17): 1822-1828.
4. Counsel SR, Callahan CM, Clark DO et al. Geriatric care management for low-income seniors – a randomized controlled trial. JAMA. 2007;298(22):2623-2633.
5. Ahmed OI, Rak DJ. Hospital readmissions among participants in a transitional case management program. Am J Manag Care. 2010;16 (10):778-783.
6. Mukamel DB, Chou C, Zimmer JG et al. The effect of accurate patient screening on cost-effectiveness of case management programs. Gerontologist 1997;37:777–784.
7. Ramsey S, Willke R, Briggs A, et al. Good research practices for cost-effectiveness analysis alongside clinical trials: The ISPOR RCT-CEA Task Force Report. Value in Health 2005; 8(5); 521-533.
8. Drummond MF, Sculpher MJ, Torrance GW et al. (2005). Methods for the economic evaluation of health care programmes. Oxford University Press, Oxford.
9. McCall N, Cromwell J. Results of the Medicare Health Support Disease-Management Pilot Program. N Engl J Med 2011; 365:1704-1712.
10. Hardcastle JD, Chamberlain JO, Robinson MH, et al.: Randomised controlled trial of faecal-occult-blood screening for colorectal cancer. Lancet 348 (9040): 1472-7, 1996.
11. REDACTED
12. Zelen M. Alternatives to classic randomized trials. Surg Clin North Am 1981; 61:1425-32.
13. MacLehose RR. A systematic review of comparisons of effect sizes derived from randomised and non-randomised studies. Hlth Tech Assess. 2000;4(34): 1-154.
14. Schwartz D and Lellouch J. Explanatory and pragmatic attitudes in therapeutic trials. J Chronic Disease, 20:637-648, 1967.
15. National Committee on Quality Assurance. HEDIS 2012, volume 2: technical specifications for health plans. Available at: http://www.ncqa.org/HEDISQualityMeasurement/HEDISMeasures/HEDIS2012.aspx. Accessed July 23, 2015.
16. Centers for Medicare & Medicaid Services. Medicare 2016 Part C & D Star Rating Technical Notes. Available at http://www.cms.gov/Medicare/Prescription-Drug-Coverage/PrescriptionDrugCovGenIn/Downloads/2016-Technical-Notes-Preview-1-v2015_08_05.pdf Accessed September 4, 2015.
17. Miller FG, Emanuel EJ. Quality-Improvement research and informed consent. NEJM. 2008;358(8): 765-767.
18. Foubister V. Quality improvement or research. Quality Matters. July/August 2008: 1-17.
19. Kass N, Pronovost PJ, Sugarman J et al. Controversy and quality improvementU: Lingering questions about ethics, oversight, and patient safety research. Joint Com J Quality Patient Safety 2008; 34(6):349-353.
20. Baily MA. Harming through protection? N Engl J Med 2008;358(8): 768-769
