# Supplementary material for: Care Coordination for High-Need, High-Cost Commercially Insured Patients: A Randomized Clinical Trial
Source: JAMA Netw Open. 2025 Jun 24;8(6):e2511804. doi: 10.1001/jamanetworkopen.2025.11804 (PMC12188368; doi:10.1001/jamanetworkopen.2025.11804)
Supplement: Supplement 2. — eTable 1. Multivariate Results Examining Cost and Utilization by Study Arm and Year eTable 2. Multivariate Results Examining Cost and Utilization by Study Arm, With Randomization Date Rather Than Engagement (or Synthetic Engagement) Date as the Index Date (n = 115 791) eTable 3. Multivariate Results Examining Cost and Utilization by Study Arm, With Dichotomous Outcomes (n = 93 379) eTable 4. Multivariate Results Examining Cost and Utilization by Study Arm, Among Patients With Continuous Enrollment Through the 12-Month Study Window (n = 52 049) eTable 5. Multivariate Results Examining Cost and Utilization by Study Arm, With a 6-Month Study Window (n = 92 838) eTable 6. Multivariate Results Examining Cost and Utilization by Study Arm, for Patients Randomized 2015 to 2017 (N = 187 791) eTable 7. Sensitivity—People Whose Study Period Did Not Overlap With the COVID-19 Pandemic (eg, Excluding March to December 2020; N = 58 466): Commercial RCT Regression Analysis Results in the 12 Months Postenrollment/Proxy, by Random Assignment Status eTable 8. Sensitivity—Commercial RCT Regression Analysis Results in the 18 Months Postenrollment/Proxy, by Random Assignment Status (N = 52 487) eTable 9. Sensitivity—Commercial RCT Regression Analysis Results in the 24 Months Postenrollment/Proxy, by Random Assignment Status (N = 53 208) eTable 10. Sample Demographics by Intervention Engagement Status [file jamanetwopen-e2511804-s002.pdf]

Supplemental Online Content

Duru OK, Harwood J, Moin T, et al. Care coordination for high-need, high-cost commercially insured patients: a randomized clinical trial. *JAMA Netw Open*. 2025;8(6):e2511804. doi:10.1001/jamanetworkopen.2025.11804

eTable 1. Multivariate Results Examining Cost and Utilization by Study Arm and Year
eTable 2. Multivariate Results Examining Cost and Utilization by Study Arm, With Randomization Date Rather Than Engagement (or Synthetic Engagement) Date as the Index Date (n = 115 791)
eTable 3. Multivariate Results Examining Cost and Utilization by Study Arm, With Dichotomous Outcomes (n = 93 379)
eTable 4. Multivariate Results Examining Cost and Utilization by Study Arm, Among Patients With Continuous Enrollment Through the 12-Month Study Window (n = 52 049)
eTable 5. Multivariate Results Examining Cost and Utilization by Study Arm, With a 6-Month Study Window (n = 92 838)
eTable 6. Multivariate Results Examining Cost and Utilization by Study Arm, for Patients Randomized 2015 to 2017 (N = 187 791)
eTable 7. Sensitivity—People Whose Study Period Did Not Overlap With the COVID-19 Pandemic (eg, Excluding March to December 2020; N = 58 466): Commercial RCT Regression Analysis Results in the 12 Months Postenrollment/Proxy, by Random Assignment Status
eTable 8. Sensitivity—Commercial RCT Regression Analysis Results in the 18 Months Postenrollment/Proxy, by Random Assignment Status (N = 52 487)
eTable 9. Sensitivity—Commercial RCT Regression Analysis Results in the 24 Months Postenrollment/Proxy, by Random Assignment Status (N = 53 208)
eTable 10. Sample Demographics by Intervention Engagement Status

This supplemental material has been provided by the authors to give readers additional information about their work.

eTable 1. Multivariate Results Examining Cost and Utilization by Study Arm and Year

Year Randomized	Outcome (Average Monthly Value)	Regression-Adjusted Mean	Δ Mean	SE	P-Value

© 2025 Duru OK et al. *JAMA Network Open*.

		Control	Treatment			
2018 (N=53,515)	Inpatient Hospitalizations	0.010	0.011	0.001	0.00	0.02
	Emergency Department Visits	0.034	0.035	0.001	0.00	0.42
	Total Plan Cost (Medical + Pharmacy)	$2,485	$2,581	$96	$56	0.09
	Medical Plan Cost	$1,337	$1,394	$58	$51	0.26
	Pharmacy Plan Cost	$1,155	$1,182	$28	$20	0.16
2019 (N=39,864)	Inpatient Hospitalizations	0.008	0.009	0.000	0.00	0.75
	Emergency Department Visits	0.031	0.031	0.000	0.00	0.72
	Total Plan Cost (Medical + Pharmacy)	$2,534	$2,552	$17	$59	0.77
	Medical Plan Cost	$1,205	$1,199	-$6	$49	0.91
	Pharmacy Plan Cost	$1,337	$1,347	$10	$30	0.73

eTable 2. Multivariate Results Examining Cost and Utilization by Study Arm, With Randomization Date Rather Than Engagement (or Synthetic Engagement) Date as the Index Date (n = 115 791)

Outcome (Average Monthly Value)	Regression-Adjusted Mean		Δ Mean	SE	P-Value
	Control	Treatment			
Inpatient Hospitalizations	0.011	0.011	0.000	0.00	0.68
Emergency Department Visits	0.036	0.036	0.000	0.00	0.96
Total Plan Cost (Medical + Pharmacy)	$2,698	$2,719	$21	$33	0.53
Medical Plan Cost	$1,451	$1,460	$9	$28	0.75
Pharmacy Plan Cost	$1,252	$1,255	$2	$15	0.87

eTable 3. Multivariate Results Examining Cost and Utilization by Study Arm, With Dichotomous Outcomes (n = 93 379)

Outcome (Any Non-Zero Value)	Regression-Adjusted Percentage		Percentage Point Change	SE	P-Value
	Control	Treatment			
Inpatient Hospitalizations	5%	5%	0%	0.00	0.26
Emergency Department Visits	16%	16%	0%	0.00	0.31
Total Plan Cost (Medical + Pharmacy)	97%	97%	0%	0.00	0.93
Medical Plan Cost	93%	93%	0%	0.00	0.96
Pharmacy Plan Cost	89%	89%	0%	0.00	0.80

eTable 4. Multivariate Results Examining Cost and Utilization by Study Arm, Among Patients With Continuous Enrollment Through the 12-Month Study Window (n = 52 049)

© 2025 Duru OK et al. *JAMA Network Open*.

Outcome (Average Monthly Value)	Regression-Adjusted Mean		Δ Mean	SE	P-Value
	Control	Treatment			
Inpatient Hospitalizations	0.006	0.007	0.001	0.00	0.02
Emergency Department Visits	0.027	0.027	0.000	0.00	0.64
Total Plan Cost (Medical + Pharmacy)	$2,224	$2,257	$33	$35	0.34
Medical Plan Cost	$1,033	$1,043	$10	$24	0.67
Pharmacy Plan Cost	$1,199	$1,209	$11	$22	0.63

eTable 5. Multivariate Results Examining Cost and Utilization by Study Arm, With a 6-Month Study Window (n = 92 838)

Outcome (Average Monthly Value)	Regression-Adjusted Mean		Δ Mean	SE	P-Value
	Control	Treatment			
Inpatient Hospitalizations	0.009	0.010	0.001	0.00	0.08
Emergency Department Visits	0.033	0.033	0.000	0.00	0.86
Total Plan Cost (Medical + Pharmacy)	$2,543	$2,596	$52	$42	0.21
Medical Plan Cost	$1,308	$1,335	$27	$37	0.47
Pharmacy Plan Cost	$1,241	$1,256	$15	$17	0.40

eTable 6. Multivariate Results Examining Cost and Utilization by Study Arm, for Patients Randomized 2015 to 2017 (N = 187 791)

Outcome (Average Monthly Value)	Regression-Adjusted Mean		Δ Mean	SE	P-Value
	Control	Treatment			
Inpatient Hospitalizations	0.015	0.015	0.000	0.00	0.59
Emergency Department Visits	0.038	0.038	0.000	0.00	0.96
Total Plan Cost (Medical + Pharmacy)	$2,866	$2,904	$38	$33	0.25
Medical Plan Cost	$1,802	$1,819	$17	$31	0.60
Pharmacy Plan Cost	$1,073	$1,082	$9	$10	0.37

eTable 7. Sensitivity—People Whose Study Period Did Not Overlap With the COVID-19 Pandemic (eg, Excluding March to December 2020; N = 58 466): Commercial RCT Regression Analysis Results in the 12 Months Postenrollment/Proxy, by Random Assignment Status

Outcome (Average Monthly Value)	Regression-Adjusted Mean		Δ Mean	SE	P-Value
	Control	Treatment			
Inpatient Hospitalizations	0.010	0.011	0.001	0.00	0.14

© 2025 Duru OK et al. *JAMA Network Open*.

Emergency Department Visits	0.036	0.036	0.000	0.00	0.91
Total Plan Cost (Medical + Pharmacy)	$2,622	$2,657	$36	$56	0.52
Medical Plan Cost	$1,423	$1,442	$20	$52	0.70
Pharmacy Plan Cost	$1,200	$1,215	$15	$19	0.43

Sample is people randomized between 2018-19 whose study period did not include the months March-December 2020 (N=58,466). Results are from linear regression. Regression covariate of interest was an indicator for randomization Group (treatment vs. control). Other covariates included sex, age group, geographic division, plan type, an indicator for whether insurance was purchased on the individual exchange, a categorical variable for the year the person was randomized (2018 vs. 2019), 19 comorbidity indicators, and a baseline measure of the outcome (for utilization variables: the average monthly value of the outcome in the 12 months before randomization; for cost variables: decile of average monthly outcome value for the 12 months before randomization).

eTable 8. Sensitivity—Commercial RCT Regression Analysis Results in the 18 Months Postenrollment/Proxy, by Random Assignment Status (N = 52 487)

Outcome (Average Monthly Value)	Regression-Adjusted Mean		Δ Mean	SE	P-Value
	Control	Treatment			
Inpatient Hospitalizations	0.006	0.007	0.001	0.00	0.04
Emergency Department Visits	0.026	0.027	0.000	0.00	0.54
Total Plan Cost (Medical + Pharmacy)	$2,166	$2,207	$41	$33	0.21
Medical Plan Cost	$984	$1,001	$17	$22	0.45
Pharmacy Plan Cost	$1,189	$1,202	$13	$22	0.57

Sample is people randomized between 2018-19 with at least one month of plan membership beyond the first 12 months post-enrollment (N=52,487). Results are from linear regression. Regression covariate of interest was an indicator for randomization Group (treatment vs. control). Other covariates included sex, age group, geographic division, plan type, an indicator for whether insurance was purchased on the individual exchange, a categorical variable for the year the person was randomized (2018 vs. 2019), 19 comorbidity indicators, and a baseline measure of the outcome (for utilization variables: the average monthly value of the outcome in the 12 months before randomization; for cost variables: decile of average monthly outcome value for the 12 months before randomization).

eTable 9. Sensitivity—Commercial RCT Regression Analysis Results in the 24 Months Postenrollment/Proxy, by Random Assignment Status (N = 53 208)

Outcome (Average Monthly Value)	Regression-Adjusted Mean		Δ Mean	SE	P-Value
	Control	Treatment			
Inpatient Hospitalizations	0.006	0.007	0.000	0.00	0.06
Emergency Department Visits	0.026	0.027	0.001	0.00	0.41
Total Plan Cost (Medical + Pharmacy)	$2,150	$2,191	$41	$32	0.20

Medical Plan Cost	$973	$987	$14	$21	0.50
Pharmacy Plan Cost	$1,184	$1,200	$15	$22	0.48

Sample is people randomized between 2018-19 with at least one month of plan membership beyond the first 12 months post-enrollment (N=53,208). Results are from linear regression. Regression covariate of interest was an indicator for randomization Group (treatment vs. control). Other covariates included sex, age group, geographic division, plan type, an indicator for whether insurance was purchased on the individual exchange, a categorical variable for the year the person was randomized (2018 vs. 2019), 19 comorbidity indicators, and a baseline measure of the outcome (for utilization variables: the average monthly value of the outcome in the 12 months before randomization; for cost variables: decile of average monthly outcome value for the 12 months before randomization).

© 2025 Duru OK et al. *JAMA Network Open*.

eTable 10. Sample Demographics by Intervention Engagement Status

	Not Engaged	Engaged (n=16,529)
	N (SD) or %	N (SD) or %
Mean age, SD	46 (12)	48 (11)
Male (n=42,786)	46	46
Female (n=50,593)	54	54
Mean months enrolled in study period, IQR	9 (6,12)	9 (6,12)
**Baseline Outcomes**		
Mean months enrolled in baseline year, IQR	9 (6,12)	9 (6,12)
Mean monthly inpatient hospitalizations, SD	0.023 (0.068)	0.023 (0.069)
Mean monthly emergency department visits, SD	0.059 (0.147)	0.062 (0.160)
Mean monthly total plan cost, SD	$3,873 ($5,792)	$3,995 ($5,834)
Mean monthly medical plan cost, SD	$2,886 ($5,675)	$2,860 ($5,616)
Mean monthly pharmacy plan cost, SD	$987 ($2,170)	$1,136 ($2,557)
**Comorbidities**		
Diabetes (n=19,852)	21	24
Arthritis (n=22,060)	23	27
Asthma (n=13,497)	14	16
Atrial Fibrillation/Cardiac Dysrhythmias (n=16,719)	18	19
Autism (n=235)	0	0
Cancer (n=31,252)	33	33
Chronic obstructive pulmonary disease (n=8,282)	8	11
Congestive heart failure (n=2,580)	3	4
Dementia (n=623)	1	1
Depression (n=18,288)	20	20
Hypertension (n=37,995)	39	46
HIV (n=2,887)	3	3
Hyperlipidemia (n=35,892)	38	42
Liver disease (n=8,289)	9	10
Myocardial infarction/Coronary artery disease	9	11
Osteoporosis (n=2,003)	2	3
Schizophrenia (n=1,042)	1	1
Stroke (n=4,445)	5	6
Substance Abuse (n=17,087)	18	19

© 2025 Duru OK et al. *JAMA Network Open*.
