# Supplementary material for: Care Coordination for High-Need, High-Cost Commercially Insured Patients: A Randomized Clinical Trial
Source: JAMA Netw Open. 2025 Jun 24;8(6):e2511804. doi: 10.1001/jamanetworkopen.2025.11804 (PMC12188368; doi:10.1001/jamanetworkopen.2025.11804)
Supplement: Supplement 3. — Data Sharing Statement [file jamanetwopen-e2511804-s003.pdf]

Data Sharing Statement

Duru. Care Coordination for High-Need, High-Cost Commercially Insured Patients. *JAMA Netw Open*. Published June 24, 2025. doi:10.1001/jamanetworkopen.2025.11804

Data
Additional Information: Trial Registration: ClinicalTrials.gov NCT04415515
Data available: No
